# Contrast-enhanced MRI of aortal atherosclerosis: syndrome types and prediction of dissection

**DOI:** 10.1186/1532-429X-17-S1-P256

**Published:** 2015-02-03

**Authors:** Alexandra S Maximova, Vadim E Babokin, Irina L Bukhovets, Yevgeniya E Bobrikova, Yuliya V Rogovskaya, Pavel I Lukyanenok, Wladimir Y Ussov

**Affiliations:** Federal State Budgetary Scientific Institution “Research Institute for Cardiology”, Tomsk, Russian Federation

## Background

Although magnetic resonance angiography is well established and deliver highly sensitive diagnosis of aneurismatic disease of aorta in patients with extensive atherosclerosis, the routine imaging of aortal wall with contrast-enhanced (CE) MRI itself is not well developed and not in routine clinical use.

## Methods

The patients population comprised 33 patients (pts, 31 men, 2 women, aged 57±9 years) with extensive atherosclerosis and old transmural acute myocardial infarction (AMI) of the left ventricle. All pts underwent cardiac CE MRI before coronary artery bypass surgery. As a control group eight patients with tumor pathology of the chest without evidence of clinically significant atherosclerosis were employed (aged 53±8 years). In addition to the CE MRI of the heart, the CE MRI of atherosclerotic lesions of the aorta was carried out. Before and in 15-20 minutes after the injection of paramagnetic contrast agent T1-weighted spin-echo images (TR=450-600 ms, TE=15 ms) were acquired. Geometric diameter and wall thickness of the aorta at the level of uptaken of contrast-paramagnetic agent to the wall, and index of enhancement (IE) were also measured.

## Results

**Figure 1:**
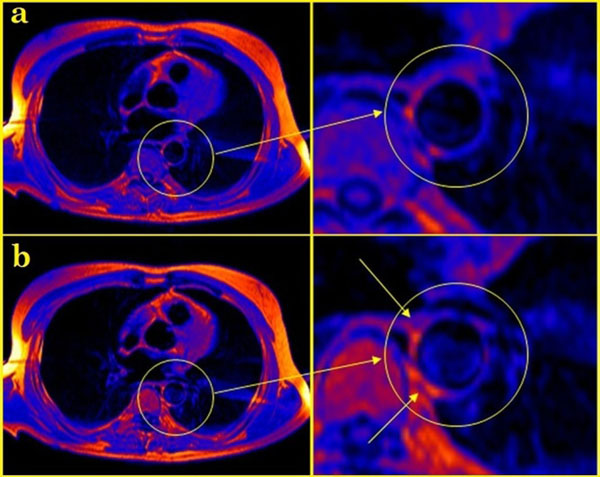
MRI in patient with extensive atherosclerosis and myocardial infarction of the left ventricular: a - T1-weighted image before contrast injection, b - T2-weighted image after contrasting - local accumulation of contrast-paramagnetic material in the aortic wall.

**Figure 2:**
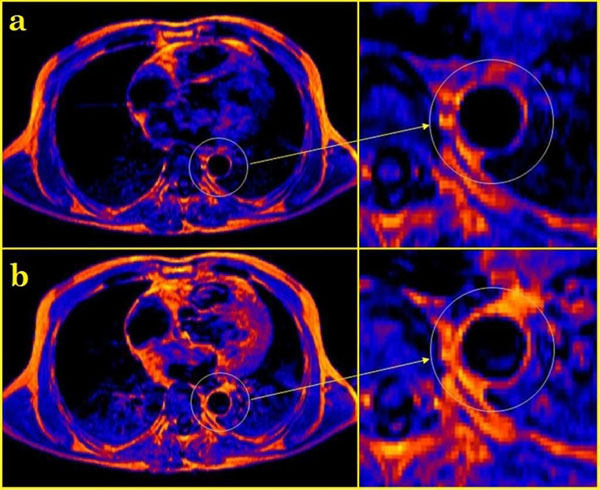
MRI in patient with extensive atherosclerosis and myocardial infarction of the left ventricular - a - T1-weighted image before contrast injection, b - T2-weighted image after contrasting - diffuse accumulation syndrome of contrast paramagnetic material in the aortic wall.

In the control group no significant accumulation of contrast paramagnetic material in the aortic wall was observed, IE did not exceed 1.04(mean 1.01±0.06), the diameter and the wall thickness at the accumulation of contrast are 2.1±0.24 cm and 0.34±0.05 cm, respectively. In 25 (76%) patients with extensive atherosclerosis and old AMI, IE of the atherosclerotic lesions in the aortic wall in all cases was over 1.14 (mean 1.17±0.13), far the over increase of intensity in the control group. In eight patients (24%) the lack of accumulation of contrast-paramagnetic material in the aortic wall was observed. Accumulation types of contrast paramagnetic were assigned to a local (Figure 1) or diffuse (Figure 2) accumulation syndrome, according to the length and circularity of the lesions. A local type of accumulation was found in 15 patients, with IE=1.09±0.06, aortic diameter and wall thickness at the level of accumulation of contrast equal to 2.66±0.35 cm and 0.5±0.13 cm, respectively. Diffuse type of accumulation was found in 10 patients; in the case the IE was as high as 1.26±0.13; aortic diameter and the wall thickness at the accumulation of contrast are 2.4±0.34 cm and 0.53±0.11 cm, respectively.

## Conclusions

MRI CE technique of atherosclerotic lesions of the aorta can be suggested as additional independent research technique in atherosclerosis prospective studies of both surgical and medical methods of anti-atherosclerotic therapy. At the moment a study of the risk of delamination, aneurysmal rupture of atherosclerotic lesions is in progress, aimed at developing additional criteria to their surgical management.

